# Multiomics Study Reveals *Enterococcus* and *Subdoligranulum* Are Beneficial to Necrotizing Enterocolitis

**DOI:** 10.3389/fmicb.2021.752102

**Published:** 2021-11-18

**Authors:** Hao Lin, Qingqing Guo, Yun Ran, Lijian Lin, Pengcheng Chen, Jianquan He, Ye Chen, Jianbo Wen

**Affiliations:** ^1^State Key Laboratory of Organ Failure Research, Guangdong Provincial Key Laboratory of Gastroenterology, Nanfang Hospital, Southern Medical University, Guangzhou, China; ^2^Shengli Clinical Medical College, Fujian Medical University, Fuzhou, China; ^3^Department of Gastroenterology, Fujian Provincial Hospital South Branch, Fuzhou, China; ^4^Department of Intensive Medicine, The First Affiliated Hospital of Fujian Medical University, Fuzhou, China; ^5^Department of Gastroenterology, Kaiping Centre Hospital, Changsha Sanjiang Development Zone, Kaiping, China; ^6^Department of Emergency, Fujian Provincial Hospital, Fujian Medical University, Fuzhou, China; ^7^Fujian Provincial Hospital South Branch, Department of Health Management, Fuzhou, China; ^8^School of Medicine, Xiamen University, Xiamen, China; ^9^Department of Gastroenterology, Integrative Microecology Center, Shenzhen Hospital, Southern Medical University, Shenzhen, China; ^10^Department of Gastroenterology, Affiliated PingXiang Hospital, Southern Medical University, Pingxiang, China

**Keywords:** NEC, FMT, sulperazone, gut microbiome, transcriptome

## Abstract

Necrotizing enterocolitis (NEC) is a life-threatening disease for premature infants with low body weight. Due to its fragile gut microbiome and successful treatment of fecal microbiota transplantation (FMT) for intestinal disease, we aimed to reveal the multiple-omics changes after FMT and/or sulperazone treatment. In this study, 2-week-old newborn rabbits were used to simulate the NEC model and grouped into healthy control, NEC, sulperazone treatment, FTM treatment, and FMT and sulperazone combination treatment. We evaluated the intestinal pathology and survival to define the benefit from each treatment and performed microbiome and transcriptome analysis to reveal the changes in microcosmic level, which could be helpful to understand the pathogenesis of NEC and develop new strategy. We found NEC rabbits benefit more from the combination of FMT and sulperazone treatment. Combination treatment reverses a lot of microorganisms dysregulated by NEC and showed the most similar transcript profiler with healthy control. Moreover, a combination of FMT and sulperazone significantly prolonged the survival of NEC rabbits. Function enrichment showed that metabolism and viral life cycle are the most significant changes in NEC. FMT is a common therapy method for NEC. Meanwhile, in the severe situation of NEC with intestinal infection, the first therapy strategy is preferred the third-generation cephalosporin, among which sulperazone is used widely and the effect is remarkable. So, we used sulperazone to treat the rabbits with the NEC. In this research, we aim to explore the different effects on NEC between FMT and sulperazone as well as the combination. Considering the microbiome and transcriptome result, we make a conclusion that the *Enterococcus* and *Subdoligranulum* benefits NEC by influencing the bacterial phages and butyrate production, respectively.

## Introduction

Necrotizing enterocolitis (NEC) is a life-threatening intestinal disease associated with an increased risk of morbidity and mortality (Papillon et al., 2017; Alganabi et al., 2019). According to epidemiological survey, about 7% of premature infants with body weights less than 1,500 g would be diagnosed as NEC (Papillon et al., 2017). However, etiologies primarily leading to the NEC are complex and unclear including premature delivery, hypoxia, and ischemia of the intestinal mucosa, infection, and gut microbiome chaos (Bi et al., 2019). Antibiotics, surgery, and advanced life support are prevailing treatments for the NEC, but the effect is limited (Bi et al., 2019).

The intestinal lumen of the newborn is considered germ-free before birth. The microorganisms from the mother’s vagina, breast milk, food, and environment will shape the gut colonized by microorganisms during the first 2 weeks of life and form the gut microbiome (Plaza-Díaz et al., 2018; Shao et al., 2019; Pilla and Suchodolski, 2020). This process of gut bacteria colonization and diversity is essential for the healthy gut of infants. Because the gut microbiome will interact with TLRs, the gut epithelial cells will develop tolerance and appropriately respond to pro-inflammatory and anti-inflammatory (Plaza-Díaz et al., 2018). The microbiome also helps to resist the pathogenic microorganisms and maintains the balance of gut microorganisms. Intestinal dysbiosis, which refers to loss of microbiome diversity and structure homeostasis in the intestine, has been proven to be associated with premature infants NEC (Papillon et al., 2017). Fecal microbiota transplantation (FMT) is an emerging and beneficial strategy to treat disease caused by gut microbiome chaos, such as chronic diarrhea, by transferring of fecal matter from healthy individuals to patients with dysbiosis to adjust the gut microbiome (Goyal et al., 2018; Antushevich, 2020; Liu et al., 2020). FMT also has been used to treat pseudomembranous colitis and shows high efficiency. Some experiments revealed that FMT is useful for infectious diseases, including NEC (Matson et al., 2018). However, the mechanisms remain to be demonstrated, especially the interactions between the microorganisms and host.

In this study, we performed gut microbiome and transcriptome analysis based on newborn rabbit models and compared among healthy, NEC, and different treatment groups. In the severe situation of NEC with intestinal infection, the first therapy strategy preferred the third-generation cephalosporin, among which sulperazone is used widely, and the effect is remarkable. So, we used sulperazone to treat rabbits with NEC. We found that NEC is characterized by metabolism dysregulation, and the FMT and sulperazone combination treatment showed the highest benefits for the NEC. The proportion of *Enterococcus* showed a significant increase after treatment, particularly after combination treatment of FMT and sulperazone. The bacterial phages carried by *Enterococcus* have been demonstrated to improve T-cell immunity, and several strains of *Enterococcus faecalis* has been proven to reverse NEC pathology (Stewart et al., 2012; Matson et al., 2018). Another significant change was the emerging of *Subdoligranulum* after combination treatment, which produces butyrate to regulate gut function (Chassard et al., 2014). These two genera may have the potential to be developed as a target for NEC treatment.

## Materials and Methods

### Animal Model and Study Design

The 2-week-old newborn rabbits (*Oryctolagus cuniculus*) were used to imitate NEC as the previous with a little change (Choi et al., 2010; Bozeman et al., 2013). Briefly, the newborn rabbits were fed with mother’s milk for 3 days before inducing NEC. The healthy controls were fed with mother’s milk all the time. The NEC rabbits were fed with homemade hypertonic formula milk (10 g protein powder dissolved in 75 ml Esbilac, 15 ml/kg/per, 3 times/day) and stimulated with hypothermia (10 min/4°C, 2 times/day for 3 days) and hypoxia (95% nitrogen and 5% oxygen. 10 L/min, 10 min/2 times/day for 3 days). The ingredients of sulperazone (cefoperazone sodium and sulbactam sodium for injection) are cefoperazone and sulbactam with a ratio of 2:1. Sulperazone was administrated to the rabbits by intravenous drip of 30 mg/kg/per 12 h. The rabbits of the groups reached 50% lethality as the end point of the experiment. The animal health condition was recorded for 2 weeks after model construction for 3 days. The animal study was reviewed and approved by the Ethics Committee of Southern Medical University (No. 2019R001-F05).

### Experimental Groups

The 2-week-old newborn suckling rabbits were selected and divided into five groups randomly, 15 rabbits for each group [Group A: healthy rabbits taken at the end of the experiment; Group B: model control group (with jugular vein venous catheter for nutrition supply); Group C: conventional treatment group: after model building successfully treated with sulperazone; Group D: FMT group (fecal bacteria transplantation treatment group): after model building successfully, healthy rabbits, they were gavaged with feces from healthy rabbits; Group E: conventional treatment and FMT group: after model building successfully, the rabbits were treated with sulperazone and FMT.

### Fecal Microbiota Transplantation

The feces of healthy baby rabbits was mixed with physiological saline, centrifuged, filtered, and the stomach was gavaged. Rabeprazole was injected before gavage to inhibit gastric acid secretion and reduce the destruction of bacterial flora in the stomach. During the experiment, fluids and antibiotics were given through the central vein.

### Intestinal Pathological Score

The intestinal tube was separated, and 2–3 cm of the ileocecum tissue was retrieved. After H&E staining, the ileocecum pathology score was performed. The pathological severity assessment was performed independently by two pathologists under double-blind conditions. The final pathology score calculation was half of the sum of the two scores. The scoring standard refers to the NADLER scoring standard: 0 points, intestinal villi and epithelium are intact, and the tissue structure is normal; 1 point, mild submucosal and/or lamina propria swelling and separation; 2 points, moderate submucosal and/or lamina propria swelling and separation, submucosal and/or muscle layer edema; 3 points, severe submucosal and/or lamina propria swelling and separation, submucosal and/or muscle layer edema, local villi loss; 4 points, intestinal villi disappearance with intestinal necrosis. The final pathology score ≥ 2 points is determined to be NEC.

### 16S rRNA Sequencing

#### DNA Extraction

Feces DNA was extracted using the Omega Stool DNA Kit following the manual. Purity and quality of the genomic DNA were checked by NanoDrop spectrophotometer (Thermo Fisher Scientific).

### PCR Amplification

The V3–4 hypervariable region of the bacterial 16S rRNA gene was amplified with the primers 338F (5′-ACTCCTACGGG AGGCAGCAG-3′) and 806R (5′-GGACTACNNGGGTATCT AAT-3′). For each feces sample, an eight-digit barcode sequence was added to the 5′ end of the forward and reverse primers (provided by Allwegene Company, Beijing). The PCR was carried out on a Mastercycler Gradient (Eppendorf, Germany) using 25-μl reaction volumes, containing 12.5 μl 2 × Taq PCR MasterMix, 3 μl of BSA (2 ng/μl), 1 μl of forward primer (5 μM), 1 μl of reverse primer (5 μM), 2 μl of template DNA, and 5.5 μl of ddH_2_O. Cycling parameters were 95°C for 5 min, followed by 28 cycles of 95°C for 45 s, 55°C for 50 s, and 72°C for 45 s with a final extension at 72°C for 10min. The PCR products were purified using an Agencourt AMPure XP Kit.

### High-Throughput Sequencing

Deep sequencing was performed on Miseq PE300 platform at Allwegene Company (Beijing). After the run, image analysis, base calling, and error estimation were performed using Illumina Analysis Pipeline Version 2.6.

### Data Analysis

The raw data were first screened, and sequences were removed from consideration if they were shorter than 230 bp, had a low-quality score (≤20), contained ambiguous bases or did not exactly match the primer sequences and barcode tags, and separated using the sample-specific barcode sequences. Qualified reads were clustered into operational taxonomic units (OTUs) at a similarity level of 97% using the Uparse algorithm of Vsearch (v2.7.1) software. The Ribosomal Database Project (RDP) Classifier tool was used to classify all sequences into different taxonomic groups against the SILVA128 database.

QIIME (v1.8.0) was used to calculate the richness and diversity indices based on the OTU information.

### RNA Sequencing

#### RNA Isolation and Qualification

Small intestine tissue RNA was extracted using the TRIzol method (TIANGEN BIOTECH, Beijing) and treated with RNase-free DNase I (TaKaRa). RNA degradation and contamination was monitored on 1% agarose gels. RNA was quantified using Agilent 2100 Bioanalyzer (Agilent Technologies, CA, United States). The quality and integrity were assessed by NanoDrop spectrophotometer (IMPLEN, CA, United States).

### Library Preparation for Transcriptome Sequencing

A total amount of 1.5 μg of RNA per sample was used as input material for the RNA sample preparations. Sequencing libraries were generated using NEBNext^®^ Ultra^TM^ RNA Library Prep Kit for Illumina^®^ (NEB, United States) following the recommendations of the manufacturer, and index codes were added to attribute sequences to each sample. Briefly, mRNA was purified from total RNA using poly-T oligo-attached magnetic beads. Fragmentation was carried out using divalent cations under elevated temperature in NEBNext First Strand Synthesis Reaction Buffer (5X). First-strand cDNA was synthesized using random hexamer primer and M-MuLV Reverse Transcriptase (RNase H). Second-strand cDNA synthesis was subsequently performed using DNA Polymerase I and RNase H. The remaining overhangs were converted into blunt ends *via* exonuclease/polymerase activities. After adenylation of the 3′ ends of DNA fragments, the NEBNext Adaptor with hairpin loop structure was ligated to prepare for hybridization. In order to select cDNA fragments of preferentially 200–250 bp in length, the library fragments were purified with AMPure XP system (Beckman Coulter, Beverly, United States). Then 3 μl of USER Enzyme (NEB, United States) was used with size-selected, adaptor-ligated cDNA at 37°C for 15 min followed by 5 min at 95°C before PCR. Then PCR was performed with Phusion High-Fidelity DNA polymerase, Universal PCR primers, and Index (X) Primer. At last, PCR products were purified (AMPure XP system), and library quality was assessed on the Agilent Bioanalyzer 2100 system. The library preparations were sequenced on an Illumina Hiseq 4000 platform by the Beijing Allwegene Technology Company Limited (Beijing, China), and paired-end 150-bp reads were generated.

### Gene Ontology Enrichment a Pathway Enrichment Analysis

Gene Ontology (GO) enrichment analysis of the differentially expressed genes (DEGs) was implemented by the GOseq R packages based on the Wallenius non-central hyper-geometric distribution, which can adjust for gene length bias in DEGs.

## Results

### Fecal Microbiota Transplantation and Sulperazone Combination Treatment Efficiently Antagonizes Necrotizing Enterocolitis

**FIGURE 1:**
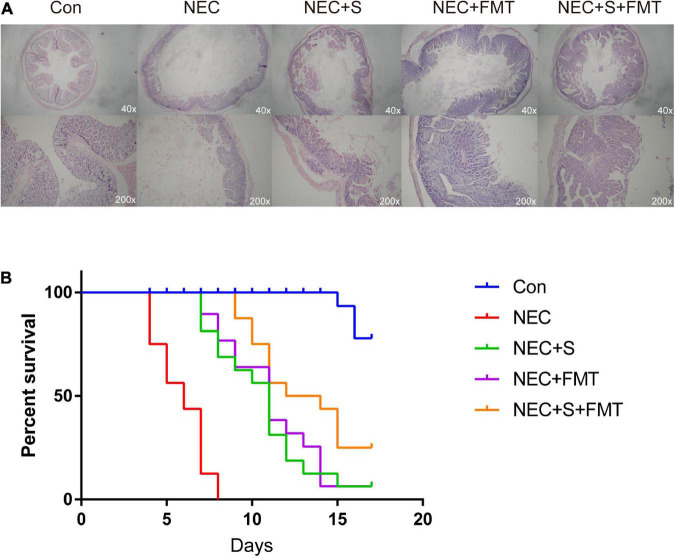
Fecal microbiota transplantation (FMT) and sulperazone combination treatment efficiently antagonizes necrotizing enterocolitis (NEC). **(A)** Pathology pictures and **(B)** survival analysis of different groups. Con, control; NEC, necrotizing enterocolitis; S, sulperazone; FMT, fecal microbiota transplantation.

Gut microbiota structure chaos is one of the leading causes of NEC. Recently, FMT is an emerging strategy to treat digestive tract disease caused by microbiome disorders. To verify whether FMT is beneficial for NEC newborn rabbits in our experiment, we constructed NEC models as described in “Materials and Methods” section, and NEC rabbits were treated with sulperazone, FMT, and its combination (Figure 1A). As shown in the figure, the NEC rabbits showed typical NEC pathology, such as intestinal mucosa destruction, villi shedding, inflammatory cell infiltration, etc. Two pathologists evaluated the sections independently, and the NEC model showed ≥ 2 score, which indicted the model had been constructed successfully. After different treatments, the pathology of NEC showed relief on different levels. However, the combined treatment of FMT and sulperazone could efficiently reverse the NEC symptom and prolong lives (Figure 1B).

### The Diversity of Microbiome Shared a Similarity With the Healthy Group After Fecal Microbiota Transplantation and Sulperazone Combination Treatment

**FIGURE 2:**
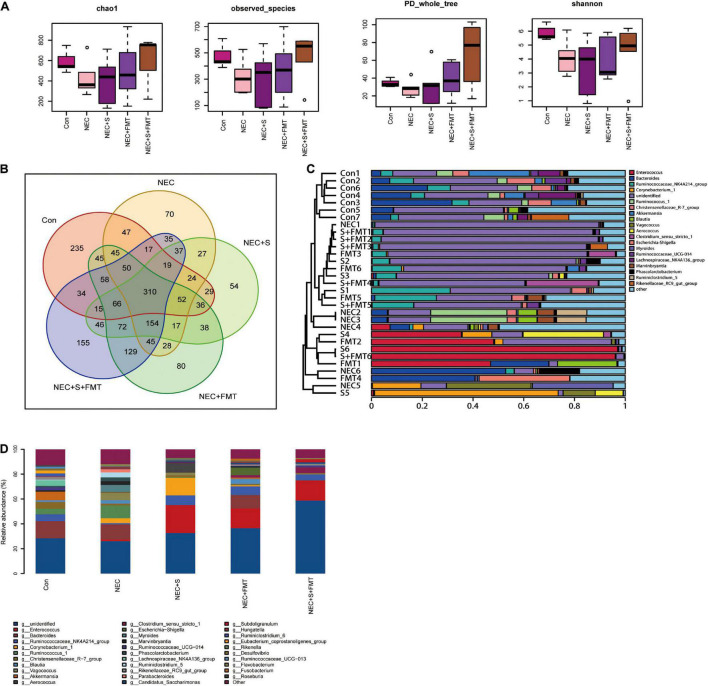
The diversity of microbiome shared a similarity to the healthy group after FMT and sulperazone combination treatment. **(A)** Four methods measured the diversity of microbiome in five groups. **(B)** Venn plot for the five groups. **(C)** Microbiome clusters of five groups. **(D)** Microbiome proportion of five groups. Con, control; NEC, necrotizing enterocolitis; S, sulperazone; FMT, fecal microbiota transplantation.

It is worth noting that the sulperazone and FMT combination shows the most efficiency (Figures 2A,B), which indicates that this therapy strategy may be a better choice for NEC treatment. We performed 16s RNA-seq to investigate how the gut microbiome changes. First, we checked the diversities of the different groups by four diversity indexes (Figure 2A). The microbiome diversity was lowest in the NEC group compared with the other groups. The microbiome diversity was significantly increased after treatments, and the combination of the sulperazone and FMT group showed the highest efficiency and shared the most similarity with the healthy group (Figures 2B,C). This result suggests that microbiome homeostasis and diversity is very important for NEC treatment, and diversity recovery is helpful for NEC treatment (Guo et al., 2021).

Then, we checked the microbiome changes on a genius level. In healthy control, *Bacteroides*, *Akkermansia*, *Ruminococcaceae NK4A214 group*, *Christensenellaceae R-7 group*, *Lachnospiraceae NK4A136 group*, and *Ruminococcus 1* were the top 10 abundant genera (Figure 2D). All the above microorganisms were probiotics, involved in food degradation, metabolism regulation, and inhibiting the pathogenic bacteria proliferation. *Ruminococcus 1*, *Marvinbryantia*, *Parabacteroides*, *Kurthia*, *Flavonifractor*, *Lactonifactor*, and *Lachnospiaceae UCG 010* were the signatures of NEC (Figure 2D; Esber et al., 2020; Hiippala et al., 2020; Lordan et al., 2020). *Parabacteroides* was reported to be upregulated in NEC (Figure 2D). The others were defined as conditional pathogenic bacteria.

Next, we compared the top five abundant microbes among different groups. *Christensenellaceae R-7 group* and *Akkermansia* were not recovered after all treatments, which may indicate that both of them were not the key microorganisms for treatment used in our experiments. *Enterococcus* and *Ruminococcaceae NK4A214 groups* were significantly increased after all treatments. The *Vagococcus* and *Myroides* were the highest in the NEC group. Moreover, they did not exist in healthy control and were downregulated by all treatments, especially by sulperazone and FMT combination, and they were discovered first in our study. The hazard of *Vagococcus* was still not clarified. However, it is clear that *Vagococcus* is associated with purulent infection (Altintas et al., 2020). *Myroides* has strong resistance to bactericides such as antibiotics and leads to infection (LaVergne et al., 2019). *Corynebacterium 1* and *Aerococcus* were the unique microorganisms of sulperazone treatment. A lot of studies reported that these two Gram-positive bacteria are conditional pathogenic bacteria (Rasmussen, 2016; Oliveira et al., 2017), and their benefits remain to be revealed. *Escherichia–Shigella* was a unique genus after FMT treatment. The abundance of *Escherichia–Shigella* was negatively correlated with NEC progress (Zhou et al., 2015). One of the mechanisms of FMT treatment to NEC was recovering *Escherichia–Shigella. Clostridium sensu stricto 1* and *Subdoligranulum* were unique genera in sulperazone and FMT combination group. *Clostridium sensu stricto 1* was helpful in promoting gut development and maintaining the gut microbiome diversity (Du et al., 2018). *Subdoligranulum* was one of the producers of butyrate, which is essential for gut health (Chassard et al., 2014). However, it could be detected in maternal feces and breast milk but does not exist in the gut of NEC babies. The combination treatment could fill the deficiency.

Interestingly, *Enterococcus* has a low proportion in NEC and healthy control but increased significantly in all treatment groups (Figures 2C,D). *Enterococcus* was classified as an infection source for inpatients and sometimes could be life threatening, but *Enterococcus faecalis*, one species of *Enterococcus*, was a common early colonizer in newborn gut and is associated with relieving NEC (Gao et al., 2018; Delaplain et al., 2019). Furthermore, a recent study showed that *Enterococcus* carries bacteriophage and interacts with human tumor antigen to enhance the T-cell immune response (Delaplain et al., 2019). Whether a similar function of *Enterococcus*, especially the *faecalis* species exists in NEC needs to be further investigated.

### The Transcriptome Shared the Similarity With the Healthy Group After Fecal Microbiota Transplantation and Sulperazone Combination Treatment

**FIGURE 3:**
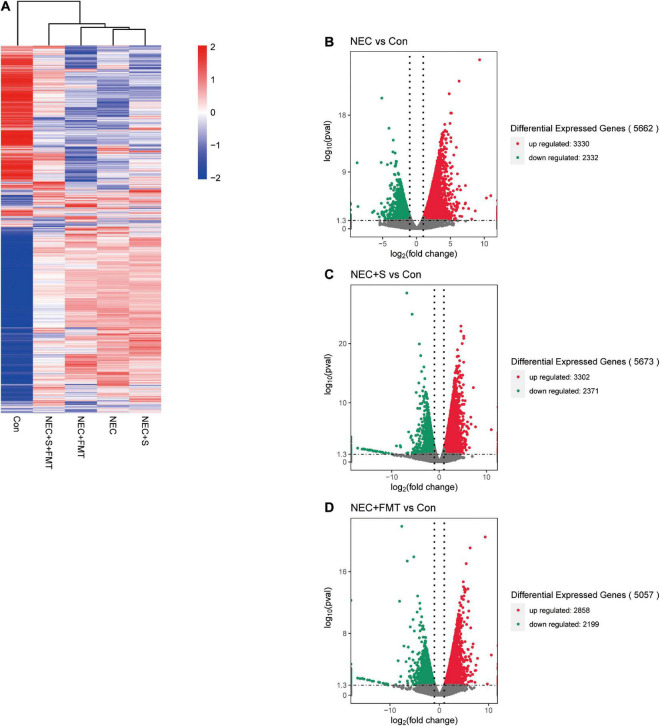
The transcription profiles in five groups. **(A)** Transcription profiles of the different groups. **(B)** Volcano plot differential expression genes between NEC and Con. **(C)** Volcano plot differential expression genes between NEC plus sulperazone and Con. **(D)** Volcano plot differential expression genes between NEC plus FMT and Con. Con, control; NEC, necrotizing enterocolitis; S, Sulperazone; FMT, fecal microbiota transplantation.

**FIGURE 4:**
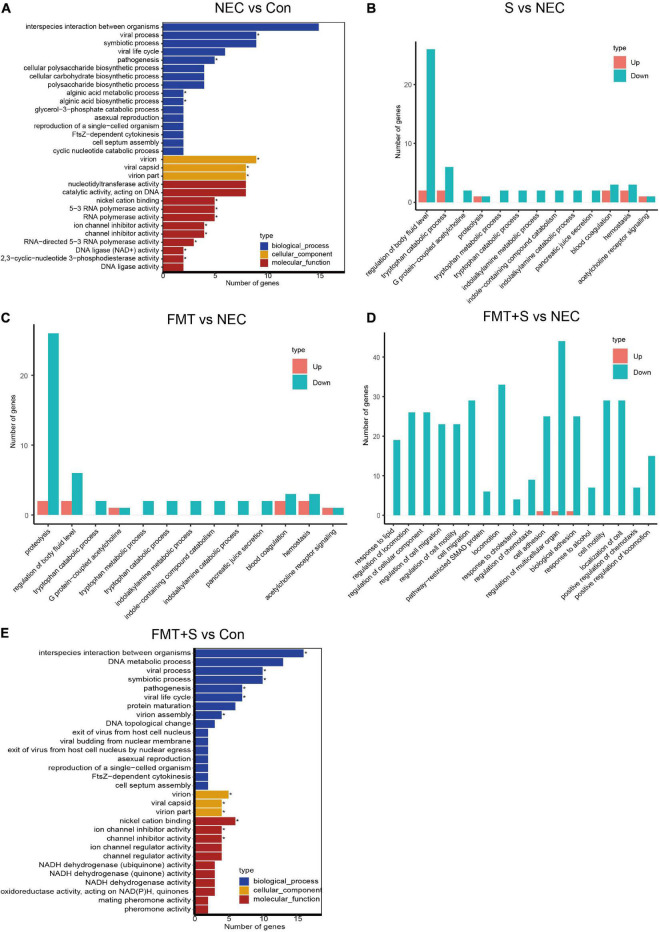
The transcriptome shared a similarity to the healthy group after FMT and sulperazone combination treatment. **(A)** GO enrichment for NEC vs. control. **(B)** GO enrichment for sulperazone vs. NEC. **(C)** GO enrichment for fecal microbiota transplantation vs. NEC. **(D)** GO enrichment for sulperazone plus fecal microbiota transplantation vs. NEC. **(E)** GO enrichment for sulperazone plus fecal microbiota transplantation vs. control. Con, control; NEC, necrotizing enterocolitis; S, Sulperazone; FMT, fecal microbiota transplantation; GO, gene ontology. *Represents for the statistic difference (*, *p* < 0.05).

To investigate gene expression change, we performed transcriptome analysis. The transcript profile of sulperazone and FMT combination treatment showed the highest similarity with the healthy group and the sulperazone treatment showed the highest similarity with the NEC group, which reminds that the combination of sulperazone and FMT may show a better benefit for NEC (Figure 3A). Then, we compared the different groups to define the further changes. We found 3,330 upregulated genes and 2,332 downregulated genes in the NEC group (Figure 3B). These genes were enriched in alginic acid metabolism, thermogenesis, oxidative phosphorylation, ribosome, and viral process, suggesting that metabolism and microbiome dysregulation were the most significant molecular changes in NEC (Figure 4A). The 3,302 upregulated genes and 2,371 downregulated genes were defined after sulperazone treatment (Figure 3C). Function enrichment analysis showed that differentially expressed genes were involved in proteolysis, tryptophan metabolism, and indolakylamine metabolism, which showed that the sulperazone may help to inhibit the necrosis (Figure 4B; Cussotto et al., 2020; Yusufu et al., 2021). Interestingly, sulperazone may inhibit the inflammation because the downregulation of tryptophan and indolakylamine could be the signature of downregulation of inflammation. FMT treatment leads to 5,057 genes changed on the expression level (2,858 upregulated and 2,199 downregulated), and they were enriched in cytokine production, proteolysis, lymphocytes and monocytes behaviors, and thermogenesis of GO (Figures 3D, 4C). Finally, we checked the pathway changes of the combination of FMT and sulperazone. Response to lipid, especially cholesterol, was the most significant downregulated pathway in metabolism, and the pathways of cell locomotion were broadly downregulated (Figure 4D).

## Discussion

Necrotizing enterocolitis is one kind of deadly disease in premature infants characterized by various injuries or damage to the intestine. The defined cause of NEC is unknown. Treatment involves stopping feedings, passing a small tube into the stomach to relieve gas, and giving intravenous fluids and antibiotics. Surgery may be needed if there is perforated or necrotic (dead) bowel tissue. About 60–80% of affected newborns survive the condition (Bi et al., 2019). FMT has showed benefits for a lot of intestinal disease by coordinating the gut microbiome (Goyal et al., 2018). Furthermore, FMT also has additional profits in metabolism and immune adjustment for patients (Lee et al., 2019).

In this study, we aimed to reveal the systematic alteration in different types with FMT and sulperazone treatment. In a multi-omics perspective, NEC was characterized with the gut microbiome and metabolism change. We found that the diversity of microbiome is declined in NEC and is reversed by FMT and/or sulperazone treatment. Interestingly, the combination of FMT and sulperazone showed the most efficiency. However, the mechanism of the results in different groups could be diversified. The FMT has the same function on benefiting the NEC by immunoregulation but from a different way. Sulperazone regulates the NEC immune response by metabolism. Sulperazone elevated microbiome diversity mainly by inhibiting harmful bacteria proliferation and showing less effects on host intrinsic behaviors, but FMT, by regulating cytokines and immune cells, was more direct and effective by supplying beneficial bacteria, and the combination treatment has both function and showed the most efficiency. Some kinds of cholesterol were important for activation of immune cells and also took part in the cell migration (Shahoei and Nelson, 2019). The combination of FMT and sulperazone may regulate the immune cell recruitment by cholesterol metabolism, and this is a different pathway to sulperazone or FMT treatment.

Intriguingly, the *Enterococcus* has a significant increase after treatment. *Enterococcus* is a genus bacterium in the intestine, which can be found under physiological conditions and could ferment carbohydrates, producing lactic acid (Liu et al., 2019). Several studies had reported that *Enterococcus faecalis*, an important species of *Enterococcus*, is decreased in NEC (Stewart et al., 2012), and some strains of *Enterococcus faecalis* is helpful in reversing the NEC pathology (Stewart et al., 2012). It should be noticed that some bacteria of the *Enterococcus* genus also carried bacterial phages, which could regulate T-cell immunity and activate cytotoxicity (Matson et al., 2018). The metabolic products of *Enterococcus* can act as feeds for other bacteria, which could help in maintaining the microbiome homeostasis. We speculate that the *Enterococcus* may have a positive function on NEC. Another genus *Subdoligranulum* is a unique genus in combination treatment, which can produce butyrate that protects the gut function. These two genera play key roles in combination treatment. We inferred based on our data that gut microbiome dysregulation leads to disorder of thermogenesis, oxidative phosphorylation, and transcription, and further metabolic disorders. The dysregulation of these pathways is consistent with the clinical symptoms of NEC. Transcriptome results show that the combination of FMT and sulperazone has the most similar transcript profile with healthy control compared with other treatment groups. Out of our expectation, the viral process is involved in NEC according to the enrichment results (Figure 4E). It may be caused by the bacteriophage activity as some reports showed that the microbes behavior could be affected by bacteriophages and further regulates the host immune response (Matson et al., 2018). All treatments could benefit NEC by metabolism and immune regulation. Sulperazone could downregulate tryptophan catabolism, and tryptophan is supposed to help to suppress the hyperinflammation (Cussotto et al., 2020; Yusufu et al., 2021). However, there are no direct evidence to conform the relationship between tryptophan metabolism and indolakylamine metabolism and sulperazone on public research, which should have a further investigation. FMT could influence cytokines production of the intestine and suppress the activation of lymphocytes and monocytes. Moreover, FMT also inhibits proteolysis like sulperazone.

In summary, we found that FMT and sulperazone combination shows the most benefits for NEC, which could significantly reverse the NEC symptoms and prolong the survival of NEC rabbits. Mechanically, the combination treatment shows the most similar transcript profiles with healthy control and could regulate the immune cell recruitment by the cholesterol metabolism. Furthermore, the gut microbe diversity is reversed in which *Enterococcus* is significantly elevated. As previously reported, *Enterococcus* faecalis showed benefits for NEC. Another is *Subdoligranulum* that can protect the gut by butyrate production. We think that *Enterococcus faecalis* and *Subdoligranulum* may have the potential to treat bacteria for NEC, and further study is needed. However, our design also has some deficiencies. The data of the different levels should be further verified by molecular experiments.

## Data Availability Statement

The datasets presented in this study can be found in online repositories. The names of the repository/repositories and accession number(s) can be found below: https://www.ncbi.nlm.nih.gov/, PRJNA753453 and PRJNA753003.

## Ethics Statement

The animal study was reviewed and approved by Ethics Committee of Southern Medical University (No.2019 R001-F05).

## Author Contributions

HL conceived, planned, wrote, and revised the manuscript. QG planned, wrote, and revised the manuscript. YR wrote and revised the manuscript. All authors have read and approved the final manuscript.

## Conflict of Interest

The authors declare that the research was conducted in the absence of any commercial or financial relationships that could be construed as a potential conflict of interest.

## Publisher’s Note

All claims expressed in this article are solely those of the authors and do not necessarily represent those of their affiliated organizations, or those of the publisher, the editors and the reviewers. Any product that may be evaluated in this article, or claim that may be made by its manufacturer, is not guaranteed or endorsed by the publisher.
